# Research Agenda for Political Science in China in an Era of Great Change

**DOI:** 10.1007/s41111-021-00191-4

**Published:** 2021-09-03

**Authors:** Guangbin Yang

**Affiliations:** Renmin University of China, Beijing, 100872 China

**Keywords:** Comparative state governance, Historical political science, Political science in China, World order, World politics

## Abstract

The world order is undergoing tumultuous changes amid the Sino–US trade war and a global pandemic. During these epochal times for political science, The American school of social sciences needs an intellectual revolution and a repositioning of the research agenda for political science. Comparative political studies must shift their focus from their traditional role of comparison of political institutions to that of state governance models, as the former can no longer advance new knowledge in political science while the latter represents a greater challenge for such studies. Likewise, studies of international relations in the traditional sense should take a step further and explore studies of world politics, i.e., studies of international relations and world order as shaped by institutional changes triggered by political trends within certain countries. The research approach of historical political science is indispensable, whether it is comparison of state governance models or of world politics.

Political science is shaped by epochal changes, hence the advent of various “neo-political studies” or “neo-political science.” Unfortunately, although not lacking in political ideology, China has been lacking the ability to set its own agenda in political science for the past hundred years. In the Republic of China, studies in this discipline followed the tradition of old institutionalism found in Europe. In the initial 30 years after the founding of the People’s Republic of China (PRC), the Soviet school played a dominant role before giving way to the American school of rational choice theory following the launch of the reform and opening-up campaign. Political science is essential in the Chinese narrative not only because the world order is undergoing profound changes and the discipline has acquired a certain degree of independence in the past hundred years in China, but also because the political practices of a world power like China require conceptualization. The “modernization of the state governance system and capacity” as a political notion based on political studies, is considered a major breakthrough in setting an agenda for political science. The profound changes to the world order will only further underscore the world-wide significance of the new agenda and showcase the need for a new agenda for the studies of international issues. The Sino–US trade war (tech war) initiated by the USA in 2018 is viewed as a milestone event in global political change that implied the self-destruction of the “free world order” dominated by the USA. Moreover, the global COVID-19 pandemic that commenced in early 2020 is widely believed to be a watershed event in the evolution of the world order. Kissinger (2020) even regards it as something that would permanently change the world order. If the Sino–US trade war (tech war) is a test field for social sciences that calls for a new research agenda, the COVID-19 pandemic has, in the least, set up a definite new agenda for political studies. This article begins with a review of the general status quo of social sciences and proceeds with a focused discussion on setting the agenda for political studies. The former provides the context for the latter.

## The State of Social Sciences: The Need of a Paradigm Revolution

As Wallerstein (2013) wrote in *The Modern World-System* (vol. 4), with the advent of a capitalist world economy, disciplines like political science, economics, and sociology that expound on the rationality of capitalism emerged. Up until World War II (WWII), more than 90% of the knowledge inputs in social sciences had been from the UK, Germany, France, the USA, and Italy and most of them focused on these five countries. International social sciences, popular after the WWII, have been dominated by the Americans. So, does the “American school of social sciences” have any intrinsic attribute? According to American scholars, the post-war American school of social sciences is actually a “study of the Cold War” (Solovey and Cravens 2021). But dissenting voices believe it is “science.” However, even if it is so, the narrative it presents is close-ended and negligent of the historical process to the effect that the end model becomes theorized. If we look at American history, we can find many staggering events, including the origin of trade protectionism, the virtual genocide of the Native Americans, etc. The state action of the USA to disintegrate Huawei will be one among such events that become history for our future generations to read. As this event unfolds, it is a rare opportunity to rethink and rebuild social sciences. Social sciences are a “generic term” that cover not only a socio-centric knowledge system, but also a state-centric one. However, the former is clearly dominant, and the ontological argument of the American school is based on the individualist hypothesis in liberalism that pervades all disciplines.

1. The political science of freedom and democracy. Political science is a discipline about the well-being of a community, but in the USA, it has been reduced to “liberal democracy,” a research paradigm, which implies that once we have established a democracy that guarantees personal rights (mainly property rights) and voting by individuals, everything would be all right.
  Its theories are based on a logic that replaces the classical notion of popular sovereignty with electoral democracy, thus equating elections with democracy and vice versa. In addition, the notion of legitimacy has been altered as well, putting forth that “legitimacy” can only be delegated through “electoral mandate,” replacing the notion itself with that of elections. Thus, “electoral democracy” and the notion of legitimacy based on it have become a criterion for the evaluation of a country’s political institution (Yi and Yang 2016). As a result, many countries were “democratized.” However, due to inefficient governance, the waves of democracy toned down into what is termed as “competitive authoritarianism” by Americans (Levitsky and Way 2010). Belief in the supremacy of freedom and democracy is deeply entrenched in the Western world so much so that, for instance, the outbreak of COVID-19 in China was viewed as a result of its institutions and that free and democratic Western countries were immune. Bias against one institution and “blind faith” in another have become shockingly deep-rooted.
  Theories on international relations based on liberal democracy-centered political science became increasingly denationalized until it reached an unprecedented level. The fall of the Berlin Wall and the collapse of the Soviet Union dealt deadly blows to the then pervasive structural realism with the state as the analytical unit. This was replaced by the liberal institutionalism founded by Fukuyama (2015) in his inspiring work *The End of History and the Last Man*. He believes that international institutions based on individualism should be regarded as universal values of a “free world order.” Accordingly, “soft power” and “democratic peace”, consistent with Fukuyama’s theory, became pervasive notions. The theory of global governance was born in this process. Rosenau (2001) believe in “governance without government” and advocate the alternative role of civil organizations. Keeping in mind the ongoing Brexit and the actions taken by Trump as the President of the United States, such notions based on liberal democracy seem rather surreal. Liberal institutionalism, claiming the USA should assimilate other countries with its “soft power,” has been proved false by the restrictive actions and violations of international regulations by Trump's administration. The US government has lost faith in its “soft power.” However, inertia prevents pervasive beliefs from withering away immediately with changes of the times.
2. Neoclassical economics based on free markets. The rise of Thatcherism and Reaganism in the 1980s set off the trend of neo-liberalism that is centered on privatization and liberalization. This political trend manifested in economics as the neoclassical school, especially as new institutional economics. It propounded that the state would prosper once the market-based ideology was established and the government protected effective property rights or private property rights. According to neoclassical economists, the key to the “rise of the West” lies in three factors: state, property rights, and ideology. However, state-building is a multi-dimensional and contextual cause. How can it depend on a few factors alone? Such theories that were based on economic history rather than actual history could be the root cause of the ineffective development of developing countries. Most developing countries adopt hereditary private land ownership as their land system. Is this an advantage or disadvantage to development? How did their performance change with land privatization? The answer can be found in what happened to Mexico since the 1980s, and the cancelation of the Jakarta–Bandung high-speed rail project in Indonesia. A glance at current world politics shows how vast the gap is between such theories and the reality. But of course, for neoclassical economists, history does not matter, and economics is all about conviction politics.
3. The sociology of “investing in people.” Governance theories driven by political democratization and economic liberalization hold that the inefficiency of their governments is the fundamental cause for the failure in development of developing countries. Therefore, they advocate reducing the borders of the state and “investing in people” to allow non-governmental organizations and individuals to take on the role of state governance. However, in the 30 years of their predominance, developing countries did not achieve progress. One reason may be that such theories confounded cause and effect. The governments of developing countries are not inefficient because they want to be so, but because they are unable to be efficient. The government is only one of the many organizations in their powerful society, and it may be overwhelmed by influential non-governmental organizations. The real mission of a developing country is to organize its different forces in national development. Advocating denationalized social rights when state power is fragmented may further undermine the already weakened state power. By adopting the aforementioned governance theories, it may worsen its own situation. The effects of “investing in people” in Western countries manifested in the critical phase of the COVID-19 outbreak as people had to depend on themselves for survival and accept “herd immunity” as a solution despite it being a very social Darwinist notion.
4. Rights-based theories of law. Jurisprudence in China, drawing on the painful lessons on violation of personal rights during the Cultural Revolution, tends to reflect on an instrumentalist approach centering on the state that advocates rights-based theories of law safeguarding human rights, since the launch of the reform and opening-up campaign. It was a giant leap forward for Chinese legal studies. However, going excessively beyond is as bad as falling short. The reason is simple: as Montesquieu wrote in “A Defense of The Spirit of Law”, law is an extension of the regime. In other words, law is primarily used to protect the regime and maintain political order. Legitimizing and then institutionalizing the political order should be the primary goal or primary political function of law. Moreover, the Roman law tradition takes the protection of private property rights as its goal, as is evidenced by natural rights and innate rights in theories of social contract. This is the economic function of law. These two functions virtually constitute the instrumentalist school of law. Lastly, the social function of law is to maintain basic security and order and protect the basic rights and interests of individuals. It is only by understanding the three functions of law that we can see the great tension between the economic system and social rights, and between the economic system and the political system safeguarded by the American constitution. Why is the country flush with guns without control even when they result in 30,000 deaths or injuries every year? Why are 40 million people not covered by medical insurance in the most developed country in the world? How can the unidimensional rights-based theories of law explain that? More importantly, the USA, the most developed and richest country in the world, suffered the largest number of COVID-19 infections while failing to effectively protect people’s lives. This could be a new conundrum for rights-based jurisprudence.
5. The American school of social sciences needs a paradigm revolution. Social sciences answer questions regarding the major issues facing a country and seek to theorize the crises or specific practices of a certain country in a specific period of history. Therefore, all social sciences must deal with historical contexts, multiple dimensions, and the extreme uncertainty and high complexity of real world problems. In one sense, theories in political science, economics, sociology, and jurisprudence that are based on liberal personal rights can be considered as efforts of justifying the results of bourgeois revolutions that advocate the protection of property rights, rather than universal doctrines applicable to the good cause of the humanity. Otherwise, Fukuyama would have seen his theories in *The End of History* come true. In fact, not only is there great tension between the aforementioned social sciences and the reality of world politics, but they are also inadequate in explaining the abuse of power by Trump’s administration. Moreover, his actions seem to have made a great mockery of prevailing social sciences. Political science, economics, sociology, and jurisprudence, as discussed previously, take “denationalization” as their fundamental goal, whereas the Trump administration represents an extreme case of power abuse. Additionally, the COVID-19 outbreak put their state capacity to test.

The Trump administration’s illegal treatment of Meng Wanzhou, the Chief Financial Officer (CFO) of Huawei, to destroy Huawei is a thorough violation of the spirit and mission of the American school of social sciences. Specifically speaking, personal rights as defined in political science are protected by law, but the arrest of Wanzhou had nothing to do with the law. The realist theories of international relations remain focused on power politics, which is one dimension of the imperialist world political structure, rather than reflecting the international relations between developed and developing countries (Yang 2020a, b). The faith in effective property rights advocated in economics was trampled by the Trump administration as it is well-known that Huawei is a typical private business. The Trump administration completely ignored the sociological hypothesis of “governance without government”, and rights-based jurisprudence was mocked worldwide during the Wanzhou incident. All these imply that the governance practices of the US government are completely contradictory to the spirit of the American school of social sciences. When theories fail to explain the reality, it means it requires reflection and rebuilding.

Thus, the American school of social sciences needs a fundamental paradigm revolution. The American school of social sciences takes denationalization as its goal. But what was its origin? Liberal social sciences are based on the hypothesis of “rational man.” Thomas Hobbes opined that the most basic element of a state is man. Therefore, to understand a state, one must analyze man first; and once “human nature” is understood, all can be solved. For Hobbes every man can be hypothesized as a “rational man” who seeks to maximize personal interests. After the WWII, social sciences, with their origin in Europe and the state as their research object, were reduced to studies of individual and social behavior, claiming the individualist hypothesis of “rational man” to be the ontological argument of all social sciences.

As is evident, such social sciences are completely ahistorical. First, they deviate from the western traditions, e.g., Aristotle’s exploration of political systems that take into account contexts and history. Second, they are inadequate to even explain the governance behavior in Western countries since they believe that the state is an indispensable dominating subject independent of the political traditions of many non-Western countries. Hence there is contradiction against the reality. Such a research paradigm that deviates from history, traditions, and reality should be replaced with a new “paradigm revolution.” The revolution, including new research approaches, should be responded in the new agenda for growth of the discipline.

## New Agenda for Comparative Politics: From Comparison of Institutions to Comparison of Governance Capacity

The post-WWII American school of comparative politics first focused on studies of political development or modernization in the 1950s–1970s and switched to those of democratization or transitology in the 1980s. Political science had a late start in China, and naturally followed the research agenda of American political science: it mainly comprised of modernization studies in the 1980s and switched to democratic transition and consolidation in the 1990s. In fact, both modernization and democratization are within the research scope of comparative studies of political institutions. This is a summary of the American school.

Of course, modernization studies can be further divided into two schools. One is the institutional school represented by Gabriel A. Almond, expressed in the form of structural functionalism. According to this line of research, once a country, irrespective of its type, acquires the seven functions extracted from the American political institution and their corresponding structure, the modernization process is successfully completed. The major dogmatic work of this school is *The Politics of the Developing Areas* published in 1959, in which all countries and regions with different cultures are analyzed in the paradigm of structural functionalism (Almond and Coleman 2017). This paradigm was prevalent in the USA until the 1970s when most of the scholars converted to the school of rational choice. Thus, we can see that methodology is prioritized by the institutional school In other words, it could be said that their methodology is designed to verify the rationality of a certain institution, which is a new approach to the relationship between methodology and political institutions. The other school of modernization studies is historical sociology represented by Barrington Moore. This school seeks to discover how modern states come into being, hence it is also known as the school of state-building. Many scholars have been conducting research on this subject, including Immanuel Wallerstein and Charles Tilly. This school enjoyed great popularity in the 1970s. Comparatively speaking, historical sociology, with history as its starting point, is less ideological. Therefore, it has left behind a richer heritage, and the discourse system created out of its knowledge system is still influencing international social sciences, especially the basic direction of political science. One major lesson that the political science discipline in China can learn from the different fates of the two schools is that knowledge in political science comes from history, and that studies of political theories originating from historical research are of historical significance even though projects rooted in historical research are more demanding. On the other hand, the methodology-prioritizing institutional schools, seeks to measure the reality with concepts or tools, but seldom produces research results that can withstand the test of time as most of its research is transient.

For reasons that are well-known, the two schools converged in the third wave of democracy in the 1980s and 1990s and both became dedicated to studies of democratization or transitology. The once-prosperous historical sociological studies of big history declined and only a few scholars are still holding on. Ideological agenda became typical of democratization studies; political science that is supposed to contribute to the “good” of state, was reduced to rhetoric; and democratization, state transformation, and other notions acquired political correctness. Thirty years after democratization studies’ ascent to popularity (1980s–2010s), American scholars began to call for “the end of the transition paradigm” and even Fukuyama admitted that what he proposed in *The End of History* should be put to an end. One major reason is that “inefficient democracy” is plaguing many countries (Carothers 2002; Fukuyama 2015).

Comparative politics, that prevailed after WWII, has served the national strategies of the USA originally. Therefore, it is inherently ideological—a trait shared by modernization and democratization studies alike (Latham 2003). However, there are differences between the two. First, democratization studies are more ideological. Among the modernization scholars, there are several historical sociologists and researchers of state-building that take knowledge contribution as their mission, whereas democratization scholars, including prestigious figures like Robert A. Dahl in his late years, have ended their pursuit of sociologically significant knowledge. Second, they have different research dimensions. Democratization studies are unidimensional as they take as their sole criterion the presence of multi-party elections. This is the underlying reason why such studies failed: as political institutions are multi-dimensional, competitive election is only the vertical form of democracy while other forms, such as deliberative democracy and participatory democracy, exist as well. Even in studies of democracy in its multiple forms, democracy is only one dimension of politics, so how can one dimension take the place of all the other dimensions of politics? Relatively, modernization studies, though ideology-oriented, are at least multi-dimensional, covering, among others, aspects, such as the development and shaping of political man, channels for attracting elites, and ways of political communication. Seymour M. Lipset, for example, takes into account the social conditions of democracy and believes that they are more important than democracy itself.

As modernization studies have become a thing of the past and democratization and paradigm of transition are declining, what research agenda or paradigm should take their place? I once suggested that comparative politics should acquire a three-in-one knowledge system encompassing “state-building—political institutions—public policies.” But of course, each research should have its specific focus. The relationship between the three can be compared to a human body: state-building is the entire body, while political institution represents the bones, and public policies the blood (Yang 2016). This reflects the prevailing comparison of institutions with transition as the paradigm, with the purpose of finding a new approach to comparative politics.

Today, this reflection seems incomplete. For instance, the comparison of state-building is more than just the relationship between war and the birth of a state from the perspective of Western historical sociology. The “nation-states” created by war are a Western case, thus, Charles Tilly built the research paradigm of “war-made states.” Many overseas Chinese scholars, including Tin-bor Victoria Hui and Zhao Dingxin in their analyses of ancient China, are practically conversing with Tilly in their studies, seeking to enrich the term with the Chinese case (Hui 2018; Zhao 2015). However, in China, war-made states only existed more than 2000 years ago. What is more intriguing is how the nation has been able to survive the 2000 years thereafter. The Chinese Empire cannot be defined by a single nation or as a “nation-state” because its history is rife with rulers of different ethnic groups. The hypothesis proposed by the school of New Qing History that Manchurians were not Chinese is false as it follows the discourse of “nation-state.” If we cannot analyze the state-building of China with theories of “nation-state,” what kind of state is China? Or what is Chineseness? A humanistic state would be a more accurate term. Whoever believes in Confucianism, a humanistic religion, can be regarded as Chinese, irrespective of their actual religion or ethnicity. In this sense, in the Chinese context, rather than looking at a “war-made state”, “state-building” entails the succession and continuity of the humanistic religion, or the contemporary significance of humanistic state. Therefore, the approach of historical political science is indispensable in comparative studies of state-building as each country’s history assumes a different political significance, and the abstract process of state-building can either be that of “war-made state” or that of “humanistic state.”

As for comparative studies of political institutions, I wrote in 比较政治学: 理论与方法 [*Comparative Politics: Theories and Methods*] that comparison of different institutions is almost extinct as studies are focusing on how states are reaching “the end of history.” I believe that, in addition to representative democracy, democratic centralism should be an equally important subject of research. Moreover, even if we need to compare political institutions, we must clarify what it means exactly and avoid taking competitive election as the sole criterion. If not, we would not be able to explain why countries that have adopted competitive elections still suffer inefficient governance or even national failure. Political science is meant to contribute to state-building and the realization of the good of a community. The organization of a community is multi-dimensional. Unidimensional political science is neither governance-oriented nor conducive to the accomplishment of the ultimate goal of the “good.”

It is more appropriate to make comparisons of state governance than to compare public policies. This is because, not only are policy making and enforcement core indicators of governance capacity, but also because governance capacity is a direct indicator of the performance of a political institution. This means that governance performance should measure whether an institution is working or not. It is often seen that the same policy can work in different ways in different countries. Governance capacity is one mediator between a policy and its effectiveness. Governance capacity can help reveal the conditional factors of policies and explain the causality better than public policies. Shifting the focus of comparative political studies from institutions to state governance capacity is not only an evolution on theoretical and conceptual levels, but also a call of the times. The global COVID-19 outbreak in early 2020 was a major test of governance capacity for all countries and a once-in-a-century case of comparative studies: as the countries experience the same event in the same period of time, their practices are comparable, unlike many previous cases that were compared despite being very far-fetched. For example, in comparative studies of institutional transition, the “institution” itself has been perceived differently—for instance, certain electoral democracies are called “competitive authoritarianism.” Similarly, in comparative studies of anti-poverty policies, the impoverished households in rural China that usually own land and property are very different from those in South Asia or South America. Their “poverty” has different connotations and is not comparable. On the contrary, the outbreak of COVID-19 has well-defined criteria, figures of infections and deaths, threat level to economy, and effects on education. As it is a common threat from a common enemy, criteria of institutional performance or governance capacity are straightforward, for instance, infection and death rates, capacity for economic recovery, and coverage of online teaching as an alternative to the traditional teaching model. In that sense, we can say that the global outbreak of COVID-19 is a once-in-a-century case for comparative political studies as it can fundamentally put an end to the “paradigm of transition” centering on institutions. If the previous calls for the “end of the transition paradigm” were voices of reason due to the bleak performance of post-transition countries, then the pandemic was a direct hit on Western countries’ faith in their institutions that had been considered as the end of history. During the initial outbreak in Wuhan, Western mainstream media attributed the tragedy to China’s non-liberal institutions, implying that free democracies could be spared of any worry. Behind the belief in institutional superiority was “white supremacy”: they even believed that the virus was contagious only among the Asians, and that the whites were immune to it. What happened later was a relentless blow to such beliefs as it was the actual governance capacity, rather than institutions, that led to different results.

The following observations were made during the outbreak: (1) countries with different institutions, e.g., China and countries like the USA and Italy, can possess different levels of governance capacity; (2) they can also possess similar levels of governance capacity, e.g., China and South Korea; and (3) countries with the same institutions can either possess the same level of governance capacity, e.g., South Korea and Germany, or they can differ in their levels of governance capacity, e.g., South Korea and Japan as compared to Italy and the USA. From such observations, it can be seen that straightforward comparisons of institutions are not necessarily effective nor are analyses of policies convincing, because the same policy can lead to different results. What can be done is to base comparative studies on the end results, i.e., governance capacity, as the most common variable, and use it to explain institutional and cultural differences. Such comparative political studies are more down-to-earth. I believe that analysis of institutions adopts a deductive method that takes one general concept as an analysis variable for studies of different countries but does not focus on the well-being of their people. Comparison of governance capacity, on the other hand, is a fact-based inductive method that compares institutional differences, functions of culture and political leadership based on actual results.

One can observe that the fundamental reason of differences in state governance capacity lies in the ability of society and state to cooperate. State–society relationship is an important approach to comparative political research, but studies in this area tend to lean towards comparative political studies with a dualist world view and emphasize society’s independence of and even antagonism towards the state. Therefore, this is considered as a subordinate method in comparative analyses of institutions. In this article, the ability of society and state to cooperate is not only a macro institution, but also a deep structure in a cultural sense. More specifically, it is a social institution and part of the fundamental structure that is directly reflected in political and even economic institutions. Irrespective of the political institution, the governance capacity tends to be greater if state and society are able to cooperate or if a tradition of cooperation exists between them; otherwise, such capacity would be weakened and beliefs in “herd immunity” or similar things would prevail. In East Asian countries and regions, including the Chinese mainland, Taiwan, South Korea, Japan, and in Germany, a strong tradition of cooperation between society and state once existed and such a tradition is termed as “state corporatism.” The economic institutions of Germany, Japan, and South Korea can be regarded as social capitalism or welfare capitalism, whereas that of China is socialist market economy. They both emphasize the coordination between politics and market. The Anglo-American countries, on the other hand, boast a liberal tradition that values individualism. Their political institution is called social corporatism and their economic institution liberal capitalism, emphasizing the decisive role of a free market. Thus, we can see that the root cause of such shared traits and differences lies in the social institution that functions as part of the fundamental structure or deep structure.

Variables that measure state governance capacity not only include state-society relationship in the sense of social institution, but also power relations as part of the political institution, and policy making and enforcement in the political process. Political institution consists of power relations and encompasses more than a fundamental political system, or regime. Regime, in turn, is manifested in the central-local relationship, political and economic relationship, ethnic relationship, inter-party relations, etc. Such a complete and multi-dimensional network of power relations may present “veto points” anytime and anywhere that can paralyze the institution and cripple state governance. Institutional integration is required to ensure the orderly and effective operation of complicated power relations. Technically, from a typological point of view, there are two types of regimes in the world: democratic centralism and representative democracy. Their capacity for institutional integration is conditioned by their specific fundamental social structure. For example, although Germany, Japan, and South Korea are all representative democracies, they differ much from the USA, the UK, and Italy in their capacity for institutional integration.

Such capacity defines the effectiveness of a country's policy enforcement. We can see that the same pandemic-control policy is enforced in very different ways in different countries. It may be due to their varied capacity for state-society cooperation, or their differing capacity for integration of political institutions. In federal countries like the USA, it is very difficult for the federal government to enforce a unified policy; and pandemic-control supplies are allocated through bidding as the belief in the market is highly entrenched. In addition, I believe that the competency and sense of purpose of policy enforcers, i.e., public servants, are a key variable for policymaking.

State governance capacity is no longer a grand and immeasurable concept, but a set of variables, the key ones being cooperation capacity that measures state-society relationship, capacity for political institution integration, and capacity for policy enforcement in the political process. This set of variables is known as “the general theory of state governance capacity,” or the research approach to or research paradigm of state governance capacity. The general features of this research paradigm are elaborated upon in previous research and are not discussed in detail in this article (Yang 2017). The general observation is that if the analysis is based on this set of variables, then, the state governance capacity is relative. No country is capable in all aspects and almost all of them face challenges or crises regarding their governance capacity, although in varying degrees. Some scholars refer to this as the “governance crisis.”

Comparative studies of state governance capacity essentially must be factual studies without any hypothesis because the relations between the variables are visible and tangible. They are comparative studies of factual results, and theories on state governance and institutional designs for governance are summed up from results. Relatively speaking, institutional comparison is more formalistic as it seeks to promote a hypothesized “good institution” and measure the entire world with the dimensions of a certain institution. It is a typical deductive method. The deductive method of comparative political science goes against the Aristotelian tradition: Aristotle, founding father of political science, summed up several types of regimes and their strengths and weaknesses through a comparative study of 158 city-states, and noted that one regime may be good in a city-state but terrible in another. The transition from institutional comparison to that of governance capacity is proof to the proposition by Samuel Huntington: countries differ not in form of government but in governance capacity (Huntington 2008). Interestingly, the transition approach of comparative political studies naturally has a bearing on the transformation of international relations studies.

## New Agenda for International Studies: From International Relations to World Politics

Earlier researches have called for the transformation from studies of international relations to world politics and demonstrated the reasons and necessity for such transformation. So, they are not discussed further in this article (Yang 2018; Yang and Shi 2020; Yao 2019, 2020). In my forthcoming book *World Political Theory*, I define world politics as world order and view international relations as shaped by domestic political reforms that are triggered by trends of political thoughts. It has the following connotations:

First, small and medium-sized countries only need to focus on interstate relations or at the most regional politics to promote their national interest, whereas for large countries, especially those with trans-regional influence, relations among great powers are important, so is world order which is a macro-level and in-depth structural issue. China, once a large yet poor country, is changing so fast that it has become a real major power. However, for those that remain large yet poor, they seem to be in no position to talk about world order: though there are ideas, they are more concerned about relations among great powers more from the point of how that can contribute more to domestic development. For real major powers, on the other hand, world order is an inevitable issue of primary concern. Judging from China’s foreign relations in the past decades, its studies of international relations and world order have been centered on itself. As studies of international relations should serve the internal development of the country, i.e., domestic economic development, relations among great powers, including Sino–US, Sino–Euro, and Sino–Japan relations, should thus be prioritized in their research agenda. World order studies serve China’s development overseas, i.e., the going-global campaign of Chinese economy and the expansion of China’s influence. China's relations with non-Western countries should be an important part of the agenda, and relevant international institutions, such as the Asian Infrastructure Investment Bank, Shanghai Cooperation Organization, and the Belt and Road Initiative, should be developed. The two lines of research represent China’s different missions in different periods of time.

Second, this definition explains the genesis of international relations and world politics in a comparative manner. As we know, studies of international relations were born out of studies of the Westphalian System. How did this system come into being? States are either at war or about to be at war, which means that war changes inter-state relations, and this forms the basis of world order. Thus, studies of international relations in this period of history focused on “power politics.” The realist notion, “balance of power,” is their main lead. The realist theories of international relations along this lead can also be correct because either the Westphalian System or the Yalta System can be considered a product of inter-state wars. It can be said that international relations are a result of external changes, such as war, and thus represent an institutional change forced by exogenous factors.

However, things changed long ago. Although war remains a common form of political intervention, at least since the end of the WWII, it is more often resorted to for maintaining the existing world order. It is difficult to construct a new order through war as it more often relies on internal changes or sudden endogenous institutional changes. The history of world politics needs rewriting. In the first round of changes, the October Revolution fundamentally changed the already existing imperialist and colonialist world system and Russia succeeded in changing the result of WWI and thus reshaping the world system by changing itself. In the second round, with the success of the Chinese Revolution, not only China was changed, but also the order of East Asia, while new nation-states launched liberation movements that resulted in the collapse of the global colonial system. In the third round, China changed itself and shook the cold-war world order by launching the reform and opening-up campaign, and the collapse of the Soviet Union marked the end of the Cold War, which caused a major shakeup of the then prevailing theories of international relations. In the ongoing fourth round, a developing China, along with the political division and veto politics resulting from identity politics in the USA, has changed the monopolar American hegemony after the Cold War.

The aforementioned domestic institutional reforms were all triggered by certain trends of political thoughts, hence they can be regarded as induced institutional change. Marxism and Leninism gave rise to the October Revolution. Likewise, the Chinese Revolution, and subsequent national independent movements were propelled by Marxism and nationalism. The third round of changes was a result of the trends of thoughts amid globalization; and the fourth round was born out of identity politics and nationalism.

Ideas shape political order, but it is regrettable that the doctrine of ideas studying political order tends to ignore the function of ideas themselves. Obviously, constructivist theories on international relations do not discuss the political thoughts that drive changes in world politics, such as socialist movements and national independence. Even those that value the relationship between ideas and foreign policies, including Robert O. Keohane, tend to confine their scope of research to the influences of ideas on individual policies, instead of adopting a macro-historical perspective on world order (Goldstein and Keohane 2005).

Theories always lag the changes of the time. However, given the great changes that have taken place, studies of international relations cannot afford to stick to the perspectives and mindsets shaped by the “old history” or ignore the “new history” that is no longer a matter of individual cases. Studying the “new history” should be one of the responsibilities of Chinese scholars towards history.

Third, the global COVID-19 pandemic constitutes a critical period for the establishment of world politics as a science. If the “new history” of idea-shaped political changes is not enough for us to value the role of ideas, this sudden event will surely shape the ideas of an entire generation, starting with a reflection on the “old history.”

According to the “old history” based on the Westphalian System, the strategic goal of national security is to look for and create enemies among countries, giving rise to a paradox: as more efforts on pursuing security can result in a greater degree of insecurity, states must build military-industrial complexes for national economy and an arms race becomes the actual primary goal of economic development. As a result, advanced technologies and weapons are developed against land, marine, air, and space enemy forces and the most advanced sectors of national economies are always related to national defense. Traditional international relations, therefore, are in what we call the (quasi-)state of war, which is a result of our dependence on the “old history.”

The “old history” cannot provide answers to questions in the “new history”, nor can it offer solutions to non-traditional threats like epidemics. Humankind has gone through several epidemics since the end of the WWII, but none of them was as deadly as COVID-19. In dealing with this threat, the national defense systems and advanced weapons that countries invested greatly in against national enemies are almost useless. Worse still, many aircraft carriers were even paralyzed by the outbreak.

The after-effects or consequences of this pandemic will be multifold. First, nationalist politics will be reinforced, which will be a retrogressive change in the process of globalization. China could become a victim of this trend, but its effects on the world order remain to be seen. Second, the effectiveness of each country’s pandemic-control measures is an indicator of its national strength and governance capacity and may lead to its repositioning in world politics. As a result, China has become a center of the thwarted globalization process. Or rather, a globalized era with China at its center will arrive earlier than expected. Thirdly, looking back at past national economies, we can see that they sought wealth and strength for national security. However, the existence of non-traditional threats, such as epidemics, requires them to adopt people's well-being as a fundamental objective and different national objectives result in different economic systems. This is also a reflection on traditional theories of state that take security and order as the primary objective of a state and see only other countries as threats. Now we can see that national security and social order are not only under the threat from other countries, but also under that from pandemics that can endanger all countries.

It is the first common threat for humankind after the WWII, and the politics that are involved in dealing with it do not entail inter-state relations any longer, but global politics in the real sense. Enemies in inter-state relations can be different for different countries. One state can be an enemy of the USA, but a friend of Russia, or a neutral existence for Europe. In global politics, however, there can only be one enemy, either an enemy of China or of other countries. As a result, if international relations seek to look for differences and pursue national security, global politics are in quest of cooperation and the security of all humankind against the existence of a common enemy. The building of a “community of shared future” has never been a need as keenly felt and as urgent as it is today.

Fourthly, there are both challenges and hopes for world politics. The pursuit of cooperation and the security of all humankind are supposed to be shared ideas for the entire generation. However, people today have lived in the “old history” and their mindset and behavior are thus constantly conditioned by the interest structure built by it. As we know, the Western world has never stepped out of the Thucydides Trap since ancient Greece, and countries are always either at war or on the brink of going to war. The process has lasted so long that a nationalist framework of thinking has taken root. Such a national psychology, once built into a people’s mind, remains highly stable and resistant to the change of times. The existing interest structure, in turn, reinforces the constructs of the history that gave rise to it. For example, the military-industrial complex of the USA supports numerous interest groups dependent on it, including military forces, industrial groups, research institutions, and the so-called think tanks. As determined by the increasing returns dynamics in path dependence, such a deep structure that dominates the destiny of the USA must maximize its interests, and seeking external “national enemies” has always been the most effective means to do so. Therefore, Huntington believes that the USA needs a moderate number of external enemies to achieve national cohesion. Thus, even though he advocates a “framework for thinking world politics,” what Huntington seeks to do is to continue to look for enemies, or “clashes of civilizations.” The “legislators” in Western history and civilizations are already too lost in their path dependence.

The fate of world politics not only depends on the status quo of the USA, but also on the development of China, because world order is shaped by the state governance capacity of countries that are at the center of the given period of time. If China becomes the center of a globalized world, its supply capacity in terms of international organizations and ideas will have major effects on world order. It is not difficult to imagine that the idea of a “community of shared future” with joint consensus, building, and sharing as principles will be of undeniable importance to the development of world order. It is worth noting that the “community of shared future” is only a natural development of humankind in history. It is a modernized rephrasing of the thousand-year-old belief in great harmony and a eulogy to the foreign policy of the People's Republic of China. It was peaceful coexistence in Mao Zedong’s era, and peace and development after reform and opening-up, followed by peaceful rising. They are all based on the recognition of the value of peace, and the building of a community of shared future will be the most ideal form of peace. Of course, “peace” has different connotations in different times. It was the pursuit of an international environment favorable to domestic development, but now it refers to advocating a new world order, completing an evolution from “inward peace” to “outward peace.”

## A Research Approach of Comparative Political Science and World Politics: Historical Political Science

A new research agenda needs a new research approach or paradigm. The approach we propose, historical political science, can be used in comparative political studies on state governance capacity, as well as in world politics (Yang 2019). Historical political science studies the structural relationalism (ontology), temporality (methodology), and contexts (epistemology) of history, extracts concepts and knowledge, and sums up principles of good governance. Historical political science is widely considered as a native Chinese school of political science, and a new approach to Chinese political science. Such a research approach that is based on local contexts particularly values findings from historical research, while world politics, by nature, is a discipline based on deep historical structure. Therefore, the two share a certain degree of affinity.

### Comparative Studies of State Governance Capacity and Historical Political Science

As discussed, comparative studies of political institutions are mainly based on a deductive logic. In the well-known book *The End of History*, Thomas Hobbes’ “rational man” hypothesis is used as the basis of the theories deducted, while the historical, cultural, and social conditions emphasized in political science are ignored. It is common sense that the same type of regime may result in quite different governance performance in different countries, such as India and the UK, or The Philippines and the USA. The inherent logic in this difference is obviously not institutional, but a matter of governance capacity.

State governance capacity is comprised of capacity for state-society cooperation, institutional integration, and policy enforcement, each of which comes from the social structure constructed over the years. Each country has an established history of civilization. Developing countries achieved political liberation only through their national independence movements and most of them did not go through a social revolution centering on land reform. Moreover, colonial heritage, i.e., fragmented politics formed out of divided rule or politics based on a strong society, is quite common. Such a social structure shaped by history tends to restrain state governance capacity in a fundamental manner.

Likewise, even for developed countries, the first-mover advantage depends on many contextual conditions. The USA, for example, made blatant genocidal attempts toward the Native Americans, plundered its colonies, and forced brazenly unequal treaties on other countries. Today, things, or at least many of them, have changed. Developing countries are catching up, fundamentally changing the historical contexts and social conditions in which the developed countries were able to have the first-mover advantage. Western and non-Western countries can be compared in terms of state governance capacity based on the same criteria. It is possible to ascertain the governance capacity of non-Western countries that adopt Western institutions and that of those that chose their own path.

Thus, historical political science is a research approach that appears to be customized for studies of state governance capacity. More importantly, historical political science, with an important role in pursuing good governance, can explain the difference in state governance capacity from a historical point of view, as well as also offer insights into state governance for each country. In fact, in each civilization, however similar the macro-level political institution may be, the middle-level institutional arrangements and micro-level political inclinations and mechanisms are always local. In that sense, by investigating middle- and micro-level institutions and behavioral patterns, we are studying historical political science about states. Each country’s governance practices and capacity, in turn, are deeply rooted in its own history. So, a better understanding of their history can help improve our understanding of their state governance capacity.

### Studies of World Politics and Historical Political Science

World politics mainly deals with international relations and world order as shaped by institutional changes within certain countries that are triggered by political trends. Here, “framework for thinking world politics” (in Huntington’s words) is first of all a deep structure constructed by history. Whether it is about history or deep structure, such research cannot do without historical political science. Moreover, world politics is a methodology based on comparative political studies and customized for comparative studies of state governance capacity. So naturally, it is applicable in world politics.

In earlier studies, I pointed out that world politics is a hierarchical concept that consists of deep structure shaped by history, status quo structure at the national level, and micro-structure at the social level. Different levels have different research units or approaches (Yang 2020a, b). The greatest concern is the deep structure shaped by history. In this line of research, there is John A. Hobson’s writing on imperialism, Immanuel Wallerstein's capitalist world-systems theory, and Huntington's theories on the clashes of civilizations. They all follow the fact-based approach of historical political science.

## Conclusion

As a research approach, historical political science is of great importance to the new research agenda. One major reason is that history is like a religion to the Chinese people. All Chinese scholars are almost born “historians.” This is not only because the Chinese civilization has a long and continued history, but also because Chinese history is mainly a political history. The historical imagination that comes therein is naturally a rare and valuable heritage for our knowledge and thinking. To a certain extent, governance thoughts and theories triggered the great changes to the world order. When the world order defined by one school of thought or theories begins to waver, there will surely be calls for new thoughts and theories. The global COVID-19 pandemic will undoubtedly contribute to this transformation. The most valuable resource that the Chinese people can offer the world is their own history of civilization and the political thoughts based on it. Historical political science is an academic and scientific form of expression for such political thoughts.
